# The Tumor Microenvironment in Classic Hodgkin’s Lymphoma in Responder and No-Responder Patients to First Line ABVD Therapy

**DOI:** 10.3390/cancers15102803

**Published:** 2023-05-17

**Authors:** Roberto Tamma, Giuseppe Ingravallo, Francesco Gaudio, Antonio d’Amati, Pierluigi Masciopinto, Emilio Bellitti, Loredana Lorusso, Tiziana Annese, Vincenzo Benagiano, Pellegrino Musto, Giorgina Specchia, Domenico Ribatti

**Affiliations:** 1 Department of Translational Biomedicine and Neuroscience, University of Bari Medical School, 70124 Bari, Italy; antonio.damati@uniba.it (A.d.); loredana.lorusso@uniba.it (L.L.); annese@lum.it (T.A.); vincenzo.benagiano@uniba.it (V.B.); 2 Department of Precision and Regenerative Medicine and Ionian Area, University of Bari Medical School, 70124 Bari, Italy; giuseppe.ingravallo@uniba.it (G.I.); francegaudio@gmail.com (F.G.); pl.masciopinto@gmail.com (P.M.); emilio.bellitti@gmail.com (E.B.); pellegrino.musto@uniba.it (P.M.); specchiagiorgina@gmail.com (G.S.); 3 Department of Medicine and Surgery, Libera Università del Mediterraneo (LUM) Giuseppe Degennaro University, 70124 Bari, Italy

**Keywords:** Hodgkin’s lymphoma, lymphocytes, macrophages, tumor microenvironment

## Abstract

**Simple Summary:**

The extracellular matrix surrounding or infiltrating tumor tissues, tumor cells, endothelial cells, immune cells, fibroblasts, macrophages, as well as soluble molecules like cytokines and growth factors secreted by these cells, constitute the complex biological structure known as the tumor microenvironment (TME). The cellular and non-cellular components of TME have a role in the development of tumors and the immune response, which offers novel insights for targeted therapies. Depleting existing cells, stopping them from being attracted to tumor locations, and reprogramming them into antitumor subtypes are the three primary categories of therapeutic approaches. TME exhibits complicated connections between Hodgkin/Reed–Sternberg cells and microenvironment and plays a crucial role in classical Hodgkin lymphoma (CHL) as well. Numerous studies have demonstrated that an extensive understanding of the CHL microenvironment, including the identification of all cellular components and variables implicated in the pathogenesis, is essential for improving prognostic stratification and developing innovative targeted treatments.

**Abstract:**

Although classical Hodgkin lymphoma (CHL) is typically curable, 15–25% of individuals eventually experience a relapse and pass away from their disease. In CHL, the cellular microenvironment is constituted by few percent of H/RS (Hodgkin/Reed–Sternberg) tumor cells surrounded from a heterogeneous infiltration of inflammatory cells. The interplay of H/RS cells with other immune cells in the microenvironment may provide novel strategies for targeted immunotherapies. In this paper we analyzed the microenvironment content in CHL patients with responsive disease (RESP) and patients with relapsed/refractory disease to treatment (REL). Our results indicate the increase of CD68^+^ and CD163^+^ macrophages, the increase of PDL-1^+^ cells and of CD34^+^ microvessels in REL patients respective to RESP patients. In contrast we also found the decrease of CD3^+^ and of CD8^+^ lymphocytes in REL patients respective to RESP patients. Finally, in REL patients our results show the positive correlation between CD68^+^ macrophages and PDL-1^+^ cells as well as a negative correlation between CD163^+^ and CD3^+^.

## 1. Introduction

Hodgkin’s lymphoma (HL) is a germinal center B-cell lymphoid neoplasm, characterized by peculiar Reed–Sternberg cells (H/RS) immersed in a wide and heterogeneous milieu. The standard diagnostic H/RS cells (15–45 μm) have at least two nuclear lobes or nuclei, are abundantly mildly basophilic, and are amphophilic in nature. At least two nucleoli must be present in two distinct nuclear lobes for HR/s cells to be diagnostic. The nuclei have a conspicuous, sometimes wavy nuclear membrane, pale chromatin, typically one prominent eosinophilic nucleolus, and a perinuclear clearing (halo) that resembles a viral inclusion. They are also large and frequently spherical in shape. The HR/S immunophenotype is CD30^+^, CD15^+^, CD45/LCA^−^, OCT2^−^ and CD3^−^ [1]. The tumor microenvironment (TME) of HL represents more than 95% of the entire tumor mass and is composed of various elements such as lymphocytes, neutrophils, eosinophils, macrophages, plasma cells, mast cells, myeloid derived suppressor cells (MDSCs), fibroblasts and endothelial cells. According to the 5th edition of WHO Classification of Hematolymphoid Tumors, HL is divided in classic HL (CHL) and nodular lymphocyte predominant HL (NLPHL) [2]. Despite the similarities between CHL and NLPHL, numerous clinicopathological and etiopathogenetic evidence demonstrated that they are almost certainly different neoplasms. CHL accounts for 10% of all lymphomas and is subdivided into four subtypes, on the basis of microenvironment composition: including the nodular sclerosis (the most common), mixed cellularity (frequently disseminated at onset), lymphocyte rich and lymphocyte depleted [3].

Most HL patients are treated by a combination of chemotherapy (adriamycin, bleomycin sulfate, vinblastine sulfate and dacarbazine, ABVD) and radiation therapy; however, 5–10% of patients will experience refractory illness after receiving first-line therapy, and 10–30% will relapse [4]. The second-line recommended treatment modality includes high-dose chemotherapy and autologous stem cell transplantation [5]. However, only a small fraction of HL patients respond in terms of long-term progression-free survival (PFS) [6]. Immune checkpoint inhibitors have altered the landscape of treatment for relapsed/refractory (R/R) CHL by enabling the rebuilding of the immune response to target malignant cells and providing a different mode of action from conventional cytotoxic chemotherapy [7]. Based on patient samples taken during and after treatment, anti-PD-1 monoclonal antibody (mAb) therapies restore the immune system’s ability to monitor cancerous cells, leading to a rapid decrease in H/RS cells and PDL1^+^ inflammatory cells as well as a long-lasting PD-1 blockade that endures even after treatment cessation [8]. Anti-PD-1 mAb monotherapy had an overall response rate (ORR) of 70% and a median of progression free survival (PFS) of 14–15 months for patients with R/R CHL who have received multiple lines of treatment, including autologous stem cell transplant (ASCT) ^+^/^−^ brentuximab vedotin (BV). Despite the fact that only 20–30% of responders in this situation achieve complete response (CR), those who do so experience a prolonged remission with a median duration of response (mDOR) of 37 months with nivolumab; additionally, mDOR was not achieved with pembrolizumab at 5-year follow-up [9,10]. In a recent study involving R/R CHL patients treated with anti-PD-1 mAb tislelizumab, 67% of patients achieved CR, and 50% of patients had ongoing response at 30-month follow-up, confirming the relationship between the depth of response and the duration of response with this class of agents [11]. In the context of numerous relapses, novel immunotherapy regimens in conjunction with other targeted treatments have increased response rates. Nivolumab (Nivo) and ipilimumab (Ipi), two checkpoint inhibitors, were combined with BV to produce CRs of 61% and 57%, respectively. However, the addition of ipilimumab led to greater toxicity than BV + Nivo in this phase I/II study, despite the fact that the combination of all three agents had the highest CR of 73%. In the BV-Ipi and triplet combination arms, the rates of grade 3–4 adverse events were observed at 43% and 50%, respectively, but only 16% in the BV–Nivo group [12]. Anti-PD-1 mAb-based treatments are therefore favored. In an early clinical study, patients with R/R CHL who had received at least two prior lines of treatment had an improvement in CR from 32% to 71% with the addition of a hypomethylating drug to an anti-PD-1 mAb, with 100% of patients continuing responding to combination therapy at a six-month follow-up. Nivolumab-JAK2 and BTK (Bruton Tyrosine Kinase) inhibitor combinations are also being tested in investigational regimens (Table 1). In a small number of patients with several relapses, retreatment with anti-PD-1 mAb has also been reported, with ORRs of 70–100% and CRs of 33–73% [13,14,15].

As these drugs are introduced to patients in earlier lines of therapy, Phase II investigation of the combination of BV/Nivo in patients who have previously received treatment with either agent is in progress and may offer more insight into the roles of sequencing and retreatment. The study of cell interactions in the TME has improved the creation of innovative treatments that either reactivate the immune system by disrupting checkpoint pathways or direct immune effector cells to attack cancerous cells in response to tumor antigens [16]. CHL may be viewed as a paradigmatic example of how the TME may influence the development of cancer. The interaction between H/RS cells and the surrounding milieu acquired a functional importance and so it has been the subject of a large amount of investigation [17]. Many experimental works demonstrated the crucial prognostic and predictive role exerted by lymphoid and monocytic compartments of the TME in CHL [18]. Different studies from our group highlighted the important roles of TME and angiogenesis in many other types of neoplasms, such as non-HLs [19,20,20,21,22] and non-hematological tumors [23,24]. TME plays a fundamental role also in CHL, and shows complex interactions between H/RS cells and microenvironment cells [24]. As demonstrated by numerous studies, a complete understanding of CHL microenvironment, with the identification of all the cellular elements and factors involved in the pathogenesis, is fundamental to better stratify prognosis and to identify novel targeted therapies. With this purpose, in our study we evaluated expression of CD34, CD68, CD163, CD3, CD8 and PD-L1 in patients with CHL, comparing the data between the groups of responders (RESP) and non-responders patients (RESP) to first-line treatment constituted by ABVD treatment [25].

## 2. Materials and Methods

### 2.1. Patients

In this retrospective investigation, bioptic samples from 30 adult patients with classical Hodgkin lymphoma (nodular sclerosing type) collected before the treatment were examined. Between 2019 and 2021, the samples were taken from the pathology section archive at the University of Bari Faculty of Medicine. Informed written consent from each patient was obtained prior to the study being conducted, and all procedures were carried out in accordance with the Helsinki Declaration of 1964 and later versions, as well as the ethical standards of the responsible committee on human experimentation (institutional and national).

The patients were divided into 2 groups based on the response to the therapy during the follow-up: 15 with responsive disease (RESP) and 15 with relapsed/refractory (REL) disease to treatment. A clear predominance of male individuals is observed in the entire sample, with a M/F ratio of approximately 2.6/1. This distribution remains preserved in the groups as well. Comparing the two populations, a significantly lower mean age was found in RESP than in REL (28.6 vs. 42.8), with a range of 24 to 34 vs. 35 to 64, respectively. In fact, this evidence showed a strong correlation, even being statistically significant at *t*-test (*p* < 0.01). In the general population the presentation of advanced (III–IV) and early (I–II) stage disease was homogeneous. No significant difference was found in the total population as well as in the two subgroups, in terms of evidence of Bulky disease, presence of B symptoms and IPS score. All individuals targeted in this study received first-line ABVD-based treatment, followed or not by radiotherapy. On the performance of the latter, there are clear differences between the two subpopulations. In fact, 83% of the responsive patients benefited from radiotherapy while only 20% of the REL individuals underwent it. In the subgroup of patients with relapsed/refractory disease to treatment, the average time elapsed to disease recurrence was 7 months, with a clear prevalence (80% of cases) of early recurrence. In most cases in which there was a rapid recurrence of lymphoma; other recurrences were observed, up to a maximum of 4. To date, no patient has gone through exitus.

### 2.2. CD3, CD8, CD68, CD163, CD34 and PDL-1 Immunohistochemistry

Serial histological sections of 4 μm thickness, collected on poly-L-lysine-coated slides (Sigma Chemical, St Louis, MO, USA), were deparaffinized. The sections were rehydrated in a xylene-graded alcohol scale and then rinsed for 10 min in 0.1 M PBS. Sections were pretreated with sodium citrate pH 6.1 solution (DAKO, Glostrup, Denmark) for antigen retrieval for 30 min at 98 °C and then incubated with mouse monoclonal anti-CD3 (DAKO, Glostrup, Denmark), mouse monoclonal anti-CD8 (DAKO, Glostrup, Denmark), mouse monoclonal anti-CD68 (DAKO, Glostrup, Denmark), mouse monoclonal anti-CD163 (DAKO, Glostrup, Denmark), mouse monoclonal anti-CD34 (DAKO, Glostrup, Denmark) and monoclonal anti-PDL-1 (DAKO, Glostrup, Denmark) diluted 1:50, 1:50, 1:100, 1:100, 1:50 and 1:100, respectively. Thereafter, the sections were counterstained with Mayer hematoxylin and mounted in synthetic medium. Specific pre-immune serum (DAKO), replacing the primary antibodies, served as negative control. The sections from each experimental group were scanned using the whole-slide morphometric analysis scanning platform Aperio Scanscope CS (Leica Biosystems, Nussloch, Germany). All the slides were scanned at the maximum available magnification (40×) and stored as digital high-resolution images on the workstation associated with the instrument. Digital slides were inspected with Aperio ImageScope v.11 software (Leica Biosystems, Nussloch, Germany) at 20× magnification and 10 fields with an equal area were selected for the analysis at 40× magnification. The protein expression was assessed with the Positive Pixel Count algorithm embedded in the Aperio ImageScope v.11 software and reported as positivity percentage, defined as the number of positively stained pixels on the total pixels in the image.

### 2.3. Statistical Analysis

Data derived from responsive disease (RESP) patients and relapsed/refractory (REL) patients are reported as means ± SE. All variables were checked for normality (Shapiro–Wilk normality test) to assess the population distribution. Unpaired two-tailed *t*-test for mean values and Pearson’s correlation coefficient for linear regression analysis were used to compare parameters with normal distribution. Data are presented as scatter dot plots with mean and standard error, with all data points shown. The Graph Pad Prism 5.0 statistical package (GraphPad Software, San Diego, CA, USA) was used for analyses and the limit for statistical significance was set at *p* < 0.05.

Correlation analysis between data derived from REL patients was performed with the Spearman non-parametric correlation test. The Graph Pad Prism 5.0 statistical package (GraphPad Software, San Diego, CA, USA) was used for analyses and the limit for statistical significance was set at *p* ˂ 0.05.

## 3. Results

### 3.1. CD68 and CD163 Immunohistochemistry

**Figure 1:**
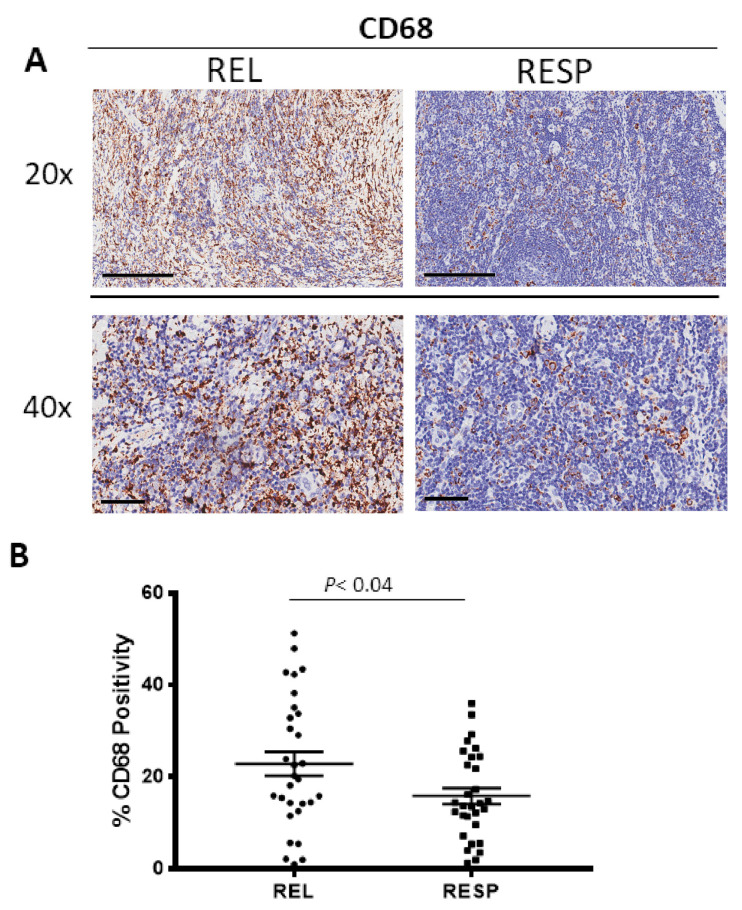
Immunohistochemical staining for CD68 in in both REL and RESP patients. Scale bar: 60 μm (40×), 200 μm (20×) (**A**). Morphometric analysis indicating the percentage of CD68 positivity in REL and RESP samples (**B**).

**Figure 2:**
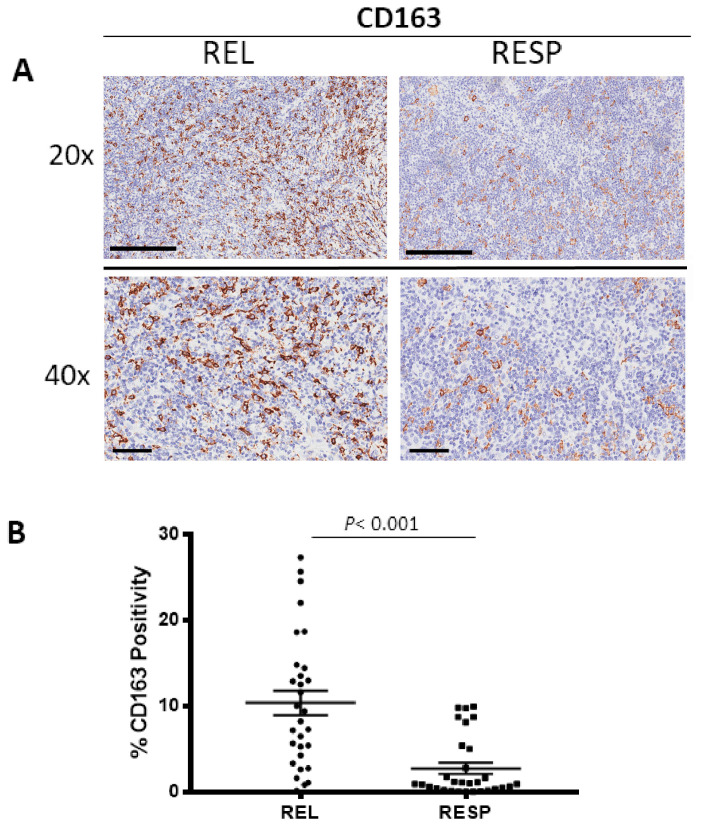
Immunohistochemical staining for CD163 in both REL and RESP patients. Scale bar: 60 μm (40×), 200 μm (20×) (**A**). Morphometric analysis indicating the percentage of CD163 positivity in REL and RESP samples (**B**).

Tissue samples derived from REL and RESP patients were immunostained for CD68 (Figure 1A) and CD163 (Figure 2A) in order to evaluate total and M2 macrophages, respectively. Morphometric analysis (Figure 1B and Figure 2B) evidences the significantly increased numbers of CD68^+^ and CD163^+^ cells in the REL tumors (CD68: 23 ± 2.6%; CD163: 10 ± 1.41%) as compared to the RESP (CD68: 16 ± 1.7%; CD163: 5 ± 1.4%).

### 3.2. CD3 and CD8 Immunohistochemistry

**Figure 3:**
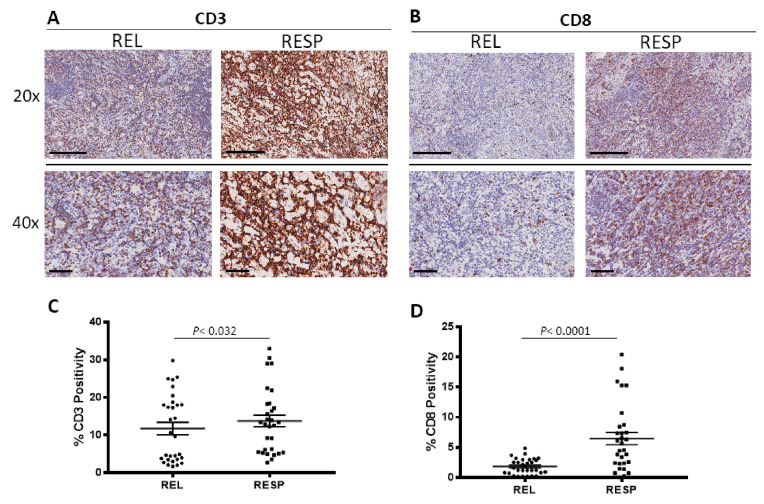
Immunohistochemical staining for CD3 (**A**) and CD8 (**B**) in in both REL and RESP patients. Scale bar: 60 μm (40×), 200 μm (20×) (**A**). Morphometric analysis indicating the percentage of CD3 (**C**) and CD8 (**D**) positivity in REL and RESP samples.

RESP and REL samples were immune stained for CD3 and CD8 to evaluate CD3^+^ (Figure 3A) and CD8^+^ lymphocytes (Figure 3B) positivity. Morphometric analysis (Figure 3C,D) indicated the significant increase of CD3^+^ and of CD8^+^ lymphocyte density in RESP (CD3: 15 ± 1.6%; CD8: 6.5 ± 1%), as compared to the REL (CD3: 10 ± 1.4%; CD8: 1.8 ± 0.24%).

### 3.3. PDL-1 Immunohistochemistry

**Figure 4:**
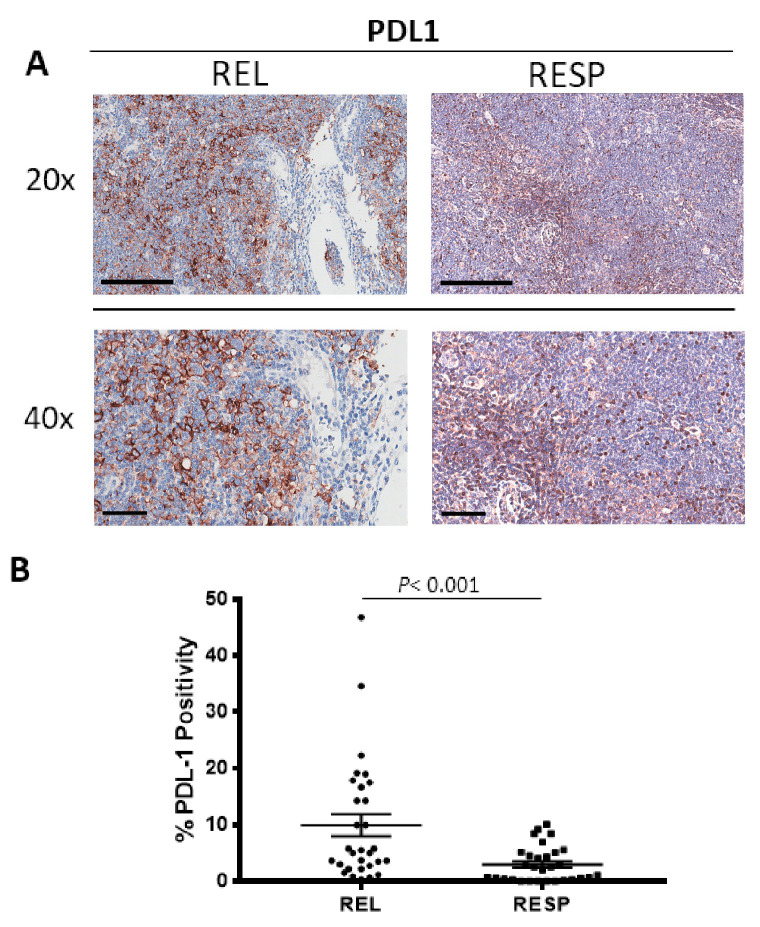
Immunohistochemical staining for PDL1 in in both REL and RESP patients. Scale bar: 60 μm (40×), 200 μm (20×) (**A**). Morphometric analysis indicating the percentage of PDL1 positivity in REL and RESP samples (**B**).

Tumor samples were immune stained for PDL-1 (Figure 4A), to estimate the PDL-1^+^ cells in the RESP and REL specimens. Morphometric analysis (Figure 4B) showed the significant increase of PDL-1^+^ positivity into the tumor REL tissue (PDL-1: 9.97 ± 1.9%) as compared to the RESP samples, RESP (PDL-1: 3 ± 0.6%).

### 3.4. CD34 Immunohistochemistry

**Figure 5:**
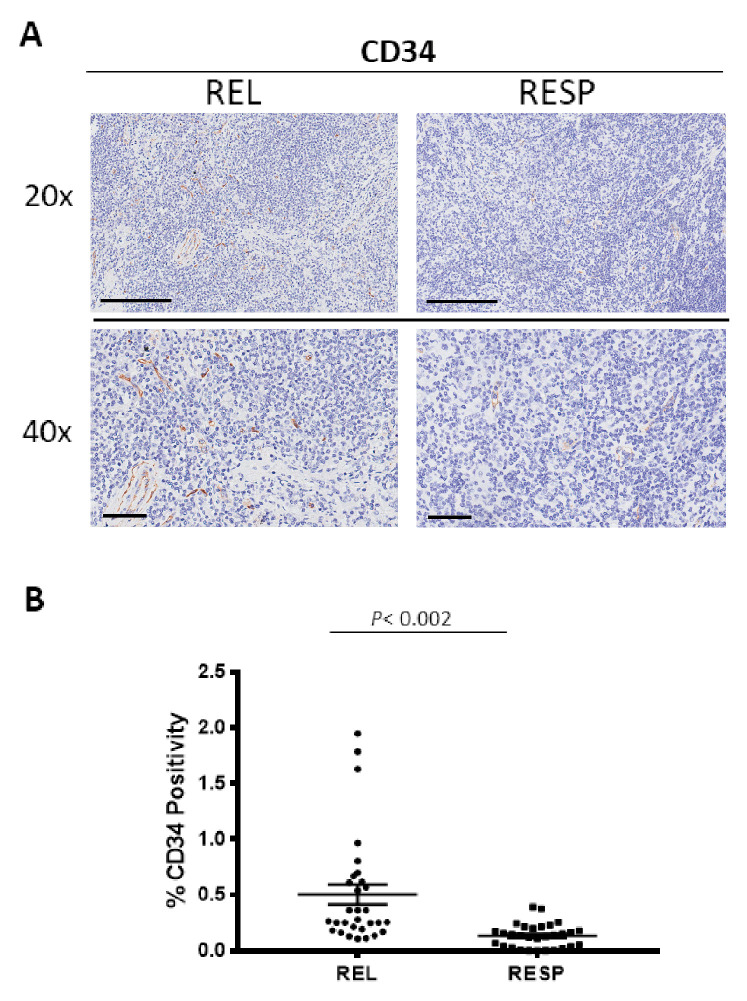
Immunohistochemical staining for CD34 in in both REL and RESP patients. Scale bar: 60 μm (40×), 200 μm (20×) (**A**). Morphometric analysis indicating the percentage of CD34 positivity in REL and RESP samples (**B**).

Tumor samples were immune stained for CD34 (Figure 5A), to estimate the microvessel density (MVD) in the RESP and REL specimens. Morphometric analysis (Figure 5B) showed the significant increase of CD34^+^ into the tumor REL tissue (CD34: 0.51 ± 0.09%) as compared to the RESP samples, RESP (CD34: 0.2 ± 0.05%).

### 3.5. Correlation Analysis

**Figure 6:**
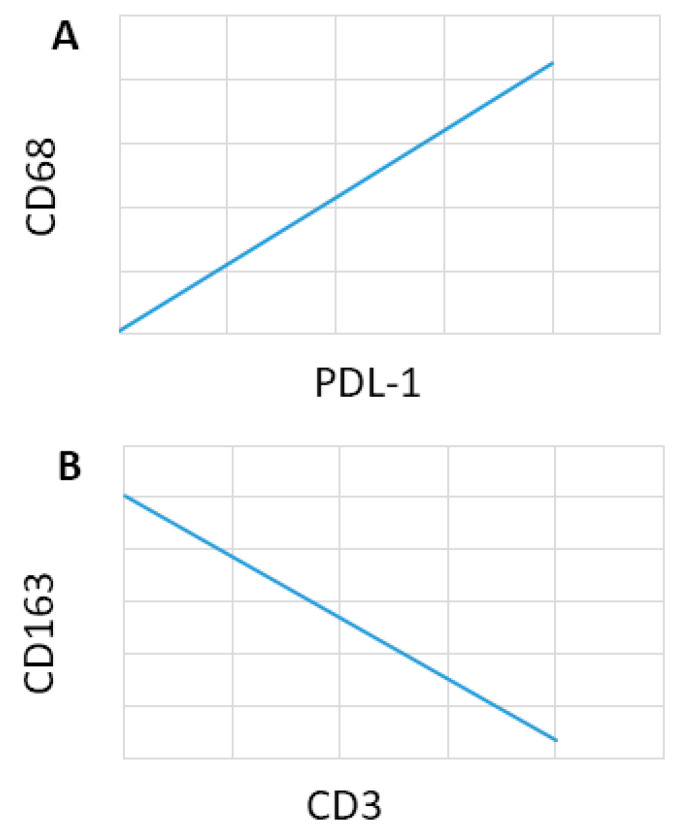
Correlation analysis in REL patients performed with the Spearman nonparametric correlation test. (**A**) positive correlation between CD68 and PDL-1; (**B**) negative correlation between CD163 and CD3.

In REL patients a positive correlation between CD68^+^ and PDL-1^+^ (rho = 0.42, *p* = 0.01) (Figure 6A), was found by Pearson’s correlation analysis. A negative correlation between CD163^+^ and CD3^+^ (rho = −0.47, *p* = 0.008) was found by Pearson’s correlation analysis (Figure 6B).

## 4. Discussion

First-line chemo-radiotherapy has a therapeutic efficacy of close to 80% in patients with advanced-stage malignancy, making CHL a chemo-sensitive disease [26]. Nevertheless, 25–30% of these patients do not benefit from the available therapies and show early disease recurrence, late disease relapse, or primary refractoriness to chemotherapy (i.e., disease progression during or within 3 months following doxorubicin-based chemotherapy) [27]. Almost 50% of late-relapsing patients with refractory/refractory CHL receive lasting responses from second-line treatment, compared to a minority of refractory individuals [28]. Patients with early recurrence and primary refractoriness respond poorly to second-line salvage chemotherapy and have extremely poor long-term disease control; therefore, their treatment constitutes an unmet medical need. New markers present in tumor cells and/or in TME enabling the early identification of chemo-refractory patients [29] or useful as targeted therapy are urgently needed. TME in CHL represents one of the most representative examples of a critical predictor of tumor development, progression and resistance to treatment in cancer [30,31]. In CHL, H/RS cells synthetize and release many factors involved in the recruitment of inflammatory cells, fibrosis induction and angiogenesis. In this context, interleukins (ILs)-5, -7, -8, -9, -13 and chemokine C–C motif ligands (CCL)-5, -17, -20, -22, recruit inflammatory cells involved in tumor growth and immune escape [32]. CHL-associated fibrosis is related to transforming growth factor beta (TGF-β), which may be directly released by H/RS cells [33,34] or by macrophages, as a consequence of IL-13 production by H/RS cells [35]. Moreover, IL-13 may also favor collagen deposition, modulating mast cell infiltration and proliferation [36]. All together, these mechanisms create a vicious circle responsible for fibrosis, which increases local concentration of cytokines and growth factors, favoring CHL progression. In this study, we have compared the CHL TME cell and microvessel content of responsive disease patients (RESP) with relapsed/refractory patients (REL). We found a decrease of CD3^+^ and of CD8^+^ T cell density in REL respective to the RESP patients. High densities of CD3^+^ and CD8^+^ cells were associated with prolonged survival and a significant therapeutic response in solid cancers [37,38]. In HL high numbers of CD8^+^ cells were associated with better outcomes, while low CD8^+^ T cell infiltrates of tumor specimens is associate with poor prognosis [39]. These T cells subsets form cluster around PD-L1^+^ cells, suggesting promotion of immunosuppression through PD-1 engagement [40]. In individuals with relapsed/refractory CHL, PD-1 inhibitors are an effective therapy strategy since 9p24.1 rearrangements in CHL are associated with PD-L1 overexpression on tumor cells in virtually all cases [41,42]. We evidenced a higher expression of PDL-1 in REL patients respective to RESP. It has been demonstrated that tumor cells inhibit the cytotoxic responses mediated by CD8^+^ T cells through the upregulation of PD-L1, which can interact with PD-1 on T cells. To transmit inhibitory signals to the T-cell receptor (TCR) pathway, PD-1 binds to PD-L1 on the surface of tumor cells and/or tumor-associated macrophages (TAMs) in the tumor microenvironment [43,44]. Cellular growth and TCR-mediated signaling activity are subsequently suppressed [45,46]. PD-1-positive TAMs may be a potential target and directly engaged in the antitumor response during immunotherapy because of the link between the PD-L1/PD-1 axis and macrophage polarization [47]. The PD-1/PD-L1 pathway is crucial to this function in tumor immunity [48]. Blocking the PD-1/PD-L1 pathway can free T cells from tumor cells’ suppressive actions and restore the T cell-mediated antitumor immune response. It has been observed that tumor CD30^+^ cells have strong expression of PD-L1 in 70–87% of CHL cases [49]. TAMs have been shown to express PD-1 and PD-L1 at a significant level. Some studies indicated a function for PD-L1 in the M1/M2 polarization and established that downregulation of PD-L1 expression was involved in the conversion from immunosuppressive to immunostimulatory macrophage phenotype [47]. In this study we demonstrated an increase of CD68^+^ and CD163^+^ TAMs in REL patients respective to RESP patients. A higher number of CD68^+^ and CD163^+^ TAMs has been correlated with shortened survival in CHL and suggests an interaction between TAMs and H/RS cells [50,51]. CD68 is an important prognostic marker in Hodgkin lymphomas and it is correlated to the poor prognosis [52]. It has demonstrated the higher CD68 expression in EBV-positive HL [53]. Mohamed et al. demonstrated that in CHL patients, CD68^+^ TAMs were related to poor response to treatment and reduced survival rates [54]. Recent findings point to a paradoxical scenario, in that TAMs may have a somewhat growth-promoting influence, and yet when their numbers increase, macrophages exhibit a growth-inhibitory effect. Finally, extremely high numbers could favor the development of tumors. These effects could also be associated with changes in M1/M2 macrophage polarization [55]. CD163 expression is associated with higher angiogenesis and shortened survival in CHL [50]. Angiogenesis is hallmark of solid and hematologic tumors and a key promoter of tumor recurrence. Since 1994, a number of studies have shown that the progression of leukemias and other hematological malignancies is strongly correlated with their degree of angiogenesis, demonstrating the significance of angiogenesis in tumor growth and metastasis [56]. Tumor angiogenesis is an important poor prognostic factor in many cancers [57,58]. Increased angiogenesis has been linked to high-grade tumors or high-grade transformation in NHLs and is associated with poor prognosis [59]. A pattern of growing angiogenesis has been demonstrated in aggressive lymphomas, as well as a statistically significant variation in total MVD across various classifications of NHL. Therefore, it was proposed that MVD may be utilized to assess angiogenic activity and aggressiveness in NHL subtypes [60]. Our results showed the increased number of CD34^+^ microvessels in REL patients respective to RESP patients. Recently, in CHL patients refractory for ABVD treatment it has been found that the upregulation of ephrin receptor signaling pathway is connected to tumor growth and metastasis and angiogenesis [61]. Correlation analyses performed in this work show a positive correlation between CD68^+^ vs PDL-1^+^ in REL patients, whereas the increase of M2 CD163^+^ macrophages is correlated to the reduction in CD3^+^ cells. M2 macrophages are attracted to tumor cells via paracrine loops or direct interaction with membrane molecules including PD-1 and PD-L1, which are crucial in the recruitment of tumor-associated macrophages (TAMs) [62]. Both carcinogenesis and the success (or failure) of anticancer treatment depend on CD68 and PD-L1. For instance, in several types of cancer, a higher CD68^+^ macrophage index is related to metastasis, a shorter time between relapses, a bad prognosis and a lower overall survival rate [63,64]. Similarly, PD-L1^+^ TAMs have been found to exist along the border between cancerous and healthy tissue. These TAMs have the potential to surround the tumor with an immunoprotective barrier, which would increase its resistance to immunotherapy. In contrast, PD-L1 inhibition reduces the pro-tumoral actions of M2 macrophages by downregulating them [62]. TAMs secrete both cytokines such as IL-10 and TGF-b which suppress the function of effector T cells and Indole 2,3-dioxygenase that lead to the depletion of tryptophan and the increase in kynurenine inhibiting clonal expansion of T cells a [65,66]. It has been demonstrated that low density of CD3^+^ cells is predictive of a poor prognosis in DLBCL [66].

## 5. Conclusions

Most of the biological and clinical properties of tumors are determined by their TME, which is by far the most prevalent component of malignancies. The description of TME in solid tumors is easy when morphology of cancer cell facilitate differentiation from immune cells. It is much more difficult in lymphoproliferative disease. Improvements in our understanding of the networks of cellular interactions in TME allowed us to recognize that, while being in the minority, the H/RS cells are the primary drivers of this dysregulated immune environment. In CHL, 5–10% of patients will experience refractory illness after receiving first-line therapy, and 10–30% will relapse. Tools that offer risk categorization (prognostic biomarkers) and, ideally, guide decisions by predicting response to various treatment regimens (predictive biomarkers) are needed to better customize treatments to individual patients.

In our retrospective study, bioptic samples from adult patients with CHL (nodular sclerosing type) collected before the treatment were examined, subdividing them into two groups: patients with responsive disease (RESP) and patients with relapsed/refractory (REL) disease. In REL patients we found the increased percentage of CD34^+^ microvessels, PDL1^+^, CD68^+^, CD163^+^ cells and the reduction of CD3^+^ and CD8^+^ cells. Moreover, the CD68^+^ cells were positively correlated to PDL1^+^ and CD163^+^ cells, but negatively correlated to CD3^+^ cells. The results led us to hypothesize that in refractory CHL patients, the immunological escape involved an increased number of macrophages, which correlates with PDL1^+^ cells, with a consequent final inhibitory immunosuppressive effect on T cells.

## Figures and Tables

**Table 1:** Clinical trials registered by the National Cancer Institute (NCI) involving combination therapies for CHL with immunotherapy drugs.

ClinicalTrial.Gov Identifier (https://clinicaltrials.gov/ct2/home)	Phase	Regimen	
NCT04866654	II	ABVD +/− PET adapted RT + Nivo in early stage non bulky disease	Frontline setting regimens
NCT03233347	II	A-AVD with PET adapted BV + Nivo; followed by Nivo maintenance in early-stage non bulky disease	
NCT03712202	II	ABVD with PET adapted BV/Nivo or ABVD in PET negative interim scan; A-AVD+ Nivo in PET positive interim scan in early-stage disease	
NCT03907488	III	A-AVD vs. Nivo-AVD in stage III/IV disease	
NCT03033914	I/II	A(B)VD +/− PET adapted Nivo-AVD in stage III/IV disease	
NCT03580408	II	Nivo +/− Vinblastine in patients aged > 60 years and unfit for standard chemotherapy in any stage disease	
NCT03739619	I/II	Nivo−Bendamustine + GVD	Combination with chemotherapy regimens
NCT04091490	II	Nivo−DHAP	
NCT03480334	II	Nivo + RT in R/R CHL	combination with radiation regimens
NCT03495713	II	Nivo + low dose RT in R/R CHL	
NCT04624984	II	PD-1 inhibitor vs. PD-1 inhibitor + GVD as first salvage	Chemotherapy sparing regimens
NCT03337919	II	Nivo monotherapy in disease refractory to 1st/2nd line salvage prior to ASCT	
NCT04938232	II	Ipi +/− Nivo in R/R cHL	
NCT04561206	II	BV + Nivo as 2nd line tx in patients not candidates for ASCT	
NCT01896999	I/II	Nivo + BV +/− Ipi in R/R CHL	Combination with other targeted therapy regimens
NCT05137886	II	Tislelizumab + Decitabine	
NCT03681561	I	Nivo + Ruxolitinib in R/R CHL	
NCT02940301	II	Nivo + Ibrutinib in R/R CHL	
NCT03057795	II	BV + Nivo consolidation post ASCT	
NCT05039073	II	patients treated with BV or CPI: BV + Nivo	

ABVD: Adriamycin, bleomycin sulfate, vinblastine sulfate, and dacarbazine; AVD: Adriamycin, vinblastine, dacabazine; ASCT: autologous stem cell transplant; B: Bleomycin; BV: Brentuximab vedotin; CR: complete response rate assessed by positron emission tomography (PET); GVD: gemcitabine, vinorelbine, doxorubicin (liposomal); Ipi: ipilimumab; Nivo: nivolumab; DHAP: dexamethasone, high dose cytarabine, cisplatin.

## Data Availability

The data can be shared up on request.
